# Disrupting Racial Dehumanization As a Root Cause of Youth Violence Through Community Engaged Visual Storytelling and Narrative Change

**DOI:** 10.1007/s11121-025-01856-1

**Published:** 2025-12-04

**Authors:** Jocelyn R. Smith Lee, Indya A. Walker, Dionna M. Tillery, Zizwe Allette, Zun Lee

**Affiliations:** 1 Department of Human Development and Family Studies, School of Health and Human Sciences, University of North Carolina, Greensboro, NC USA; 2 Centering Black Voices Research Lab, Department of Human Development and Family Studies, School of Health and Human Sciences, University of North Carolina, Greensboro, NC USA; 3 Youth Opportunity (YO!) Baltimore Program, Historic East Baltimore Community Action Coalition (HEBCAC), Baltimore, MD USA; 4 College of Communication & Information Sciences, University of Alabama, Tuscaloosa, AL USA

**Keywords:** Youth violence, Black youth, Structural racism, Firearm violence, Violence prevention

## Abstract

In 2020, firearm violence became the leading cause of death for American children and teens, a critical datapoint informing the 2024 U.S. Surgeon General’s advisory on firearm violence. However, firearm violence has been a leading cause of death for Black youth—particularly, Black males—for decades, disproportionately impacting their morbidity and mortality. As the rights of Black youth to experience safety from firearm violence converge with the interests of white youth now increasingly impacted by it, it is imperative that prevention scientists critically interrogate what contributes to the national willfulness to see Black youth as perpetrators of violence deserving of punishment and a national reluctance to see Black youth as victims of violence deserving of healing and prevention? Guided by the Cycle of Dehumanization framework, we contend this pattern is symptomatic of racial dehumanization, a root cause of structural racism and violence. We argue that to successfully prevent youth firearm violence using a structural approach, we must disrupt dehumanizing narratives about Black male criminality and offer a viable solution through our visual storytelling and narrative change campaign, *In All Ways Human*. Using an adapted community engaged participatory action research approach and qualitative interviewing, our multimodal narrative change project captured 50 strategically disseminated (mural, billboards, kiosks, exhibits, digital galleries) portraits and stories that construct a counter-narrative with the power to prevent youth violence by transforming the ways in which Black males are seen, see one another, and see themselves. The impact and future directions of our narrative change effort are discussed.

“The most difficult social problem in the matter of Negro health is the peculiar attitude of the nation toward the well-being of the race. There have, for instance, been few other cases in the history of civilized people where human suffering has been viewed with such peculiar indifference.” . W.E.B. Du Bois, 1899, The Philadelphia Negro.

In 2020, firearm violence became the leading cause of death for American children and teens (Kegler et al., 2022), a critical inflection point informing the U.S. Surgeon General’s advisory on firearm violence (United States Department of Health & Human Services, 2024). However, firearm violence has been a leading cause of death for Black youth—Black boys and young men, in particular—for decades, disproportionately impacting their morbidity and mortality (Centers for Disease Control and Prevention, 2024; Villareal et al., 2024). Black youth are disproportionately vulnerable to nonfatal (fights, penetrating violence) and fatal violent injuries than white youth (Sheats et al., 2018). The gun fatality rate for Black children and teens is over 18 times higher than the gun fatality rate for white youth (Villarreal et al., 2024). Black male youth and young adults (ages 15–34) are 20 times more likely to die from firearm violence than their white male peers (Centers for Disease Control and Prevention, 2024). These health inequities prematurely claim the lives of Black youth in the U.S., depriving their families, communities, and society of their presence, talents, and contributions, and buffeting their families with trauma and grief in its wake (Smith Lee & Patton, 2016; Sharpe, Bailey, & Richardson, 2024).

Black male youth (ages 18–24) in a Baltimore-based qualitative study examining the unequal burdens of traumatic loss and bereavement resulting from homicide (Smith, 2015), on average, suffered the loss of three loved ones to homicide. Homicide victims overwhelmingly mirrored their peer survivors in age, gender, race, and socioeconomic status, highlighting the intersectional vulnerability to violence exposure for Black youth residing, grieving, and attempting to heal in neighborhoods of concentrated disadvantage. As Buggs et al. (2023) contend in their empirical review of the structural and social determinants of community firearm violence and community trauma, the structuring of susceptibility to firearm violence and safety from it is not randomized, but racialized. It is the fruit of centuries of structural racism in which white Americans leveraged “mutually reinforcing inequitable systems (in housing, education, employment, earnings, benefits, credit, media, health care, criminal justice, etc.)” (Bailey et al., 2017, p. 1453) to codify the power structure of white supremacy and construct a racial hierarchy that systematically advantages white people and systematically disadvantages Black people. Vulnerability to firearm violence is no exception; conversely, it is exemplar (Sharpe, Bailey, & Richardson, 2024).

Recent research is beginning to empirically examine the connections between anti-Black structural racism and firearm violence (Mehranbod et al., 2022). Mehranbod and colleagues (2022) estimated the relationship between historical redlining by the federal Home Owners’ Loan Corporation (HOLC)–a governmental praxis of grading and mapping the desirability and risk of city neighborhoods for government-sponsored loans, with the neighborhoods with the highest proportions of Black residents being circled in red as least worthy of HOLC investment–and contemporary firearm violence in 21 American cities. For 17 of 21 cities, redlined zip codes were generally associated with higher rates of firearm deaths. Of interest to the locale of this project, the examined effects were significantly stronger in the city of Baltimore evidencing the empirical relationship between structural racism and the incidence of firearm violence.

Uzzi et al. (2023) extended this research by examining historical (redlining) and contemporary (economic disadvantage) dimensions of structural racism and nonfatal shooting rates in Baltimore. Using an intersectional approach, their goal was to investigate how two dimensions of structural racism, past and present, would be associated with nonfatal shootings in this city. Study findings indicated that Baltimore census tracts that experienced both contemporary economic disadvantage and historical HOLC redlining–conceptualized as sustained disadvantage tracts–suffered the highest rates of nonfatal shootings; and, this additive interaction explained over 33% of nonfatal shootings 2015–2019.

Despite public health research making a strong case that racial disparities in firearm violence are symptomatic of/maintained by the social determinants of health (SDOH) (Buggs et al., 2023) and associated with structural racism (Mehranbod et al., 2022; Uzzi et al., 2023), the public discourse around youth violence largely remains individually focused, with Black male victims often perceived as deserving of the victimization they suffer (Rich, 2009; Sered, 2015). Adapted from Neal’s (2013) work on Black masculinities and theorized as legible (real, clearly visible, and sensical in the national imagination) or conversely illegible, Leonard (2017) argues that racial narratives about space, place, and firearm violence shape public perceptions, media responses, and policy decisions about the inevitability of violence, the visibility of pain, the innocence of victims, and the interventions that are marshalled (or not) in response. As such, community firearm violence impacting the lives of Black Americans in economically disadvantaged urban contexts is typed common, expected, insular, and thus self-inflicted and therefore illegible and unworthy of national concern (Leonard, 2017). Whereas firearm violence such as mass shootings are typed atypical, unexpected, suburban, random, tragic, and narrowly—and inaccurately—perceived as exclusively happening to white Americans and therefore legible and worthy of national concern. Likewise, Leonard (2017) examined media and political discourse following firearm violence events and observed the patterned query of the lives of white perpetrators of firearm violence as a search for explanations of their unimaginable actions (Leonard, 2013), in contrast to the patterned search for criminality across the lives of Black firearm violence victims to substantiate assumptions about the deservedness of their own deaths.

In his powerful article, Gregory Jackson, Jr., former Deputy Director of the first ever White House Office of Gun Violence Prevention, wove his personal story of being shot and the presumptions of culpability he experienced in the aftermath into a larger narrative of the ways Black communities are vilified and blamed for their own suffering by the public and policy makers (Jackson, Jr., 2022). For Jackson, Jr., the failure to perceive Black firearm victims–who are most vulnerable to experiencing the toll of gun violence–as innocent (Goff, Jackson, Di Leon, Culotta, & DiTomass, 2014; Leonard, 2017), exacerbated his own pain while simultaneously rendering it invisible–a duality of dehumanization that denied his claim to empathy and a shared humanity.

While there is no singular definition of dehumanization, at its core, dehumanization is “the denial of full humanness to others, and the cruelty and suffering that accompany it” (Haslam, 2006, p. 252). Despite documenting the health inequities of youth violence that produce unequal burdens of trauma (Smith & Patton, 2016), loss (Smith, 2015), grief (Sharpe, et al., 2024), and vulnerability to recurrent violent injury (Richardson et al., 2016), our nation has been slow to acknowledge and address the pain firearm violence creates in the lives of Black youth and the structural violence that maintains it (Farmer, 2004; Jackson & Saddler, 2022); a seeming continuation of the *peculiar indifference* Du Bois penned in 1899 (Loren Dreier, 2021). In contrast, law, policy, and public narrative have been swift to target firearm violence as a crime committed by Black youth, marshalling a criminogenic response that exacerbates racial disparities in the carceral state (Leonard, 2017; Sered, 2015).

As the rights of Black youth to experience safety from firearm violence converge with the interests of white youth (Crenshaw, Gotanda, Peller, & Thomas, 1995) now increasingly impacted by it, it is imperative that prevention scientists critically interrogate what contributes to our national willfulness to predominantly see Black youth as perpetrators of violence deserving of punishment and our national reluctance to see Black youth as victims of violence deserving of healing and prevention. Guided by the *Cycle of Dehumanization* framework (Forward Promise, 2019), we argue that the failure to see and systematically respond to the pain of Black youth resulting from firearm violence is symptomatic of racial dehumanization, a root cause of structural violence and racism (Jones, 2000). In this paper, we argue that to successfully and sustainably address the health inequity of youth firearm violence using a structural approach, we must name the failure to fully see Black youth as human beings worthy of safety, care, and opportunity as a critical challenge in the work of youth violence prevention. We offer an innovative and viable community-engaged intervention strategy for multi-level youth violence prevention that disrupts dehumanizing racial narratives, *In All Ways Human*.

## Literature Review

Between me and the other world is an ever unasked question: Unasked by some through feelings of delicacy; by others through the difficulty of rightly framing it. All, nevertheless, flutter round it…How does it feel to be a problem? W.E.B. Du Bois, 1903.

I would love to have Gov. [Wes] Moore call, because I watched him over the weekend trying to explain, ‘Baltimore, what we need is housing.’ No, they don’t need housing. They need to get rid of the criminals. These are hard-core criminals. You know, we took many people off the streets of Washington, D.C. They’re hardcore. They’re not going to be good in 10 years, in five years, in 20 years, in two years they’re going to be criminals. They were born to be criminals.

Frankly, they were born to be criminals.

47th President of the United States, September 2025, White House.

## Racial Dehumanization, the Narrative of Black Male Criminality, and Violence

Although the praxis of dehumanization is centuries old and has been the focus of critical essays, creative works, and theorizing across disciplines (Fanon, 1961; McKittrick, 2015), the empirical cannon examining dehumanization is relatively young, emerging as the focus of psychological research and theory in the early 2000s (Haslam, 2006; Haslam & Stratemeyer, 2016). According to their review of recent dehumanization research (Haslam & Stratemeyer, 2016), the experiences of racial-ethnic minority groups in the U.S. (African Americans) and internationally (Arabs, Palestinians, Roma) are central to this scholarship, with an empirical focus on understanding intergroup relations, contributing factors and consequences of dehumanization for minoritized groups, and prevention. As examined by Haslam (2006) in his integrative theoretical review of dehumanization, the denial of humanity is conceptually patterned as two major forms: (1) Denial of uniquely human characteristics (e.g. civility, refinement, moral sensibility, rationality/logic, and maturity), and (2) denial of human nature (e.g. emotional responsiveness, interpersonal warmth, cognitive openness, agency/individuality, depth). Haslam (2006) named the first form of dehumanization *animalistic*, where people who are denied uniquely human characteristics are perceived, compared to, and treated as if they are amoral and animal-like. Haslam (2006) typed the second form of dehumanization *mechanistic*, where people are perceived, compared to, and treated as if they are “soulless machines” (p. 258). In both forms, those dehumanized are considered subhuman through the denial of humanity.

Animalistic dehumanization is foundational to the construction of the dominant negative narrative of Black male criminality (BMC). In his sociological work, *Black Image in the White Mind: The Debate on Afro-American Character and Destiny, 1817–1914*, sociologist George M. Frederickson (1971) details the ways in which wealthy white Americans (men and women) leveraged the power of narrative and storytelling to justify their fictitious hierarchy of humanity, the brutality of chattel slavery, Jim Crow laws, and racial terror lynchings. In his chapter, “The negro as beast: Southern Negrophobia at the turn of the century,” Frederickson mapped out the narrative shift from casting enslaved Africans as *docile* to emancipated Africans as *degenerate* and dangerous to justify the continued pursuit of white dominance post-emancipation.

As made clear by Frederickson’s (1971) historical analysis, this strategic narrative shift was advanced through religious books (1900: *The Negro a Beast* by clergyman Charles Carroll; 1903: *The Leopard’s Spots* by Baptist minister Thomas Dixon), literary works (1905: *The Clansman* by Thomas Dixon), film (1915: *Birth of a Nation* by D.W. Griffith), scientific racism in medicine (1903: Negro’s susceptibility to “sexual madness” published in *Medicine* by Baltimore physician, Dr. William Lee Howard) and social science (1899: “Negro by nature was a criminal type” p. 281—Walter F. Wilcox, address to the American Sociological Association), and legal pamphlets (1891: Baltimore lawyer William Cabell Bruce and Baltimore clergyman and 1899: Philip Alexander Bruce; brothers) promulgated at the turn of the century, heightening racial anxieties in the wake of “unsupervised” communities of freed Black Americans. The most widely recognized of this propaganda, *Birth of a Nation*, was the first film to be screened at the White House evidencing the national uptake of this negative narrative. In cities like Baltimore, this dominant cultural mindset informed policy, with the first-of-its-kind residential segregation ordinance being passed in 1910 (Boger, 2009; Power, 1983), setting the national stage for structural racism (Bailey et al., 2017) and structural violence (Jackson & Saddler, 2022) in the forms of legal racial segregation, redlining, police violence (Smith Lee & Robinson, 2019) and the entrenched social forces that constitute contemporary SDOH (Buggs et al., 2023).

The dehumanizing animalistic storytelling of Black males as *brutes* and *beasts*, who are prone to crime and sexually prey upon white women if they are not controlled and physically dominated by white men, often visually depicted Black men as brute apes (e.g. Beauty and the Beast; King Kong). Goff and colleagues specifically examined the consequences of this historical ‘Black/ape association as a mechanism of animalistic dehumanization for Black boys and men among white male college students (Goff, Eberhardt, Williams, & Jackson, 2008) and police officers (Goff, Jackson, Di Leone, Culotta, & DiTomasso, 2014). In “Not yet human,” Goff et al. (2008) found evidence that the Black-ape metaphor implicitly influenced racial bias among a sample of white male undergraduates. When this association was activated through lab testing, participants perceived hypothetical Black suspects as more deserving of excessive force by police officers in a video task than hypothetical white suspects for the same alleged crimes.

In their 2014 paper, “The essence of innocence,” Goff et al. extended this research by exploring the dehumanization of Black children and Black boys with a sample that included both college students and police officers. The researchers were interested in whether Black children and boys were perceived as older, less innocent, and thus more culpable of crime, responsible for their actions, and deserving of punishment. Study findings evidenced the continued influence of the Black-ape metaphor and racial bias, the overestimation of age for Black children and boys by college students and law enforcement officers, and the role of dehumanization in denying protective assumptions of innocence in childhood to Black children, especially boys, who are perceived as older and held to a higher standard offering insight into these racial disparities.

Psychological research has also empirically examined a *superhuman* form of dehumanization as experienced by African Americans, in particular (Waytz, Hoffman, & Trawalter, 2015). Superhumanization is defined as “the attribution of supernatural, extrasensory, and marginal mental and physical qualities to humans” (Waytz et al., 2015, p. 352). Rather than being perceived and treated as subhuman, persons who are superhumanized are regarded as more than human. Research examining superhumanization offers empirical evidence that people (both white and Black) implicitly assume that Black people feel less physical pain (Trawalter, Hoffman, & Watyz, 2012). Watyz and colleagues (2015) extended Trawalter’s work and found both implicit and explicit evidence of the superhumanization bias among their sample of all white participants, with participants more likely to explicitly endorse that an injured Black male was less likely to need pain medication than an injured White male with the same injury.

Even in death, Black males who suffer a violent death at the hands of police are dehumanized (Leonard, 2017; Smiley & Fakunle, 2016). In their paper, Smiley and Fakunle (2016) reviewed the continued evolution of the Black male criminality narrative from historic depictions of *brute* to contemporary characterizations of *thug*. Specifically, they examined this linguistic strategy within media descriptions of unarmed Black male victims as a mechanism of posthumous dehumanization in cases of officer-involved killings. Their content analysis of news articles revealed the ways in which media descriptions of six highly publicized cases of police killings in 2014–2015 (Eric Garner, Michael Brown, Jr., Akai Gurley, Tamir Rice, Tony Robinson, Freddie Gray) de-victimized Black boys, youth, and men through media storytelling about victims’ behavior, appearance, location, and lifestyle. These findings foreground the life course nature of racial dehumanization that extends from the womb to the tomb. Taken together, the empirical evidence on dehumanization offers critical insights into theorizing about the interconnectedness of the dehumanizing narrative of Black male criminality and the systematic denial of pain to Black male victims of youth firearm violence and their perceived deservedness and resulting implications for addressing youth violence prevention from a structural approach.

## The Cycle of Dehumanization, Narrative of Black Male Criminality, and Youth Violence

In 2019, Forward Promise, an initiative funded by the Robert Wood Johnson Foundation, that centers on reclaiming humanity as a priority in cultivating communities where youth of color can thrive, launched its Dehumanization Framework that “identifies dehumanization as the foundation for systemic racism, racial trauma, and toxic stress for people of color both in historical and contemporary contexts.” In their *Cycle of Dehumanization* model, Forward Promise theorized dehumanization as a vicious cycle. Specifically, they identified the following four core reinforcing elements: (1). Negative narratives about people of color; (2). Dangerous actions toward people of color; (3). Harmful internal feelings of people of color; and (4). Destructive external reactions of people of color; reactions that are typically pathologized and thus cited to reinforce the negative narratives about people of color named in this cycle. In this cyclical model, negative narratives about people of color are the bedrock upon which structural racism and structural violence are built. Thus, disrupting these narratives holds tremendous power to dismantle racism, advance healing, and marshal sustained youth violence prevention and intervention efforts.

When we examine the *Cycle of Dehumanization* (COD) model from a youth violence prevention lens, the need to disrupt the dehumanizing narrative of Black male criminality is clear and essential. The narrative of Black male criminality (COD negative narratives) undergirds structural racism and structural violence like systematic disinvestment from communities of color (COD dangerous actions), which begets the social conditions (SDOH) that increase the propensity for youth violence. These conditions are not randomized but racialized, rendering Black youth, males in particular, at disproportionate risk for youth violence exposure (COD harmful internal feelings and harmful external reactions). However, the narrative of Black male criminality shrouds the visibility of Black male vulnerability and victimization to youth violence. Instead of mobilizing investments in healing responses, this health inequity reinforces the negative narrative of Black male criminality; funneling public and political responses towards a criminal justice versus a public health approach, and the COD repeats. Therefore, if we are going to take a structural approach to preventing youth violence, we argue that critical investments must be made in narrative change work. In the following sections, we describe what narrative change is, provide key definitions that guide this space of intervention, and offer a viable solution to prevent youth violence through our innovative visual storytelling and narrative change campaign, *In All Ways Human*.

## Narrative Change and Youth Violence Prevention

Recognizing the power of narrative for youth violence prevention creates a pathway of intervention that addresses the structural underpinnings and maintenance of this health inequity for Black males. Narratives have been conceptualized by both their format (structure) and function (purpose) (Korobkova et al., 2023). While format emphasizes narratives as patterns or a collection of stories that represent core ideas held by both individuals and groups, narratives function as a mechanism to make meaning of such stories, shaping how we see the world (Lynn & Kathlene, 2020). Narratives are built and refined over time, circulating at various levels including interpersonally and institutionally. Narratives often reflect dominant ideology, reinforcing the beliefs and values of the majority (Han & Kalra, 2024). A dominant narrative is defined as the “most prominent and prevalent ways of depicting an issue, topic, or group” (FrameWorks Institute, 2024, p. 7). The dominant negative narrative of Black male criminality is an example of stories (e.g. “tales about particular events and people; may be true or fictional” FrameWorks Institute, 2024, p. 5) repeated and widely accepted, that have shaped the mindsets of the public from the past to present.

Narrative change is defined as “a change in the narratives that circulate within public discourse: either in the set of narratives in circulation (i.e. which narratives are used) or in their relative prevalence (i.e. which narratives are used more or less frequently), or both” (Han & Kalra, 2024, p. 4). The term narrative change is sometimes used interchangeably with the term narrative shift strategy, both of which capture the “collective, extended efforts to broadly reshape public discourse, thinking, and decisions on a particular topic” (FrameWorks Institute, 2024, p. 8). The growing field of narrative change aims to not only challenge harmful narratives that normalize inequity, but to replace them with new, counter-narratives that shift mindsets and activate long-term structural change (Forward Promise, 2019; Kalra et al., 2021). Counter-narratives “resist a dominant narrative by providing a different way of depicting an issue or group” (FrameWorks Institute, 2024, p. 7). Counter-narratives can be emergent (e.g. #BlackLivesMatter) and/or cultivated – “developed deliberately and strategically by advocates, activists, or other change makers” (FrameWorks Institute, 2024, p. 7). Our work aims to prevent youth violence by disrupting the narrative of Black male criminality through the creation and dissemination of a counter-narrative centering Black male humanity with the potential to shift public mindsets and policy decisions about Black boys and men and violence prevention.

Though narrative change is an emerging and evolving multidisciplinary endeavor, there are four emergent insights from the field: (1) there is no fixed formula; instead, narrative change is context-specific (Lynn & Kathlene, 2020); (2) there are various approaches; narrative change involves decision-making about whose voices are centered, which tools are used, and where impact and reach are desired (Lynn & Kathlene, 2020); (3) there are various sectors and systems in which narrative change operates including “mass media, mass culture, and mass movements” (Moore & Sen, 2022, p. 22); 4) there is no linear path; instead, narrative change is a dynamic process that includes strategy, storytelling, testing, and refining along the way (Jenkins, 2018).

While there is limited empirical research on narrative change and mindset shifts (Han & Kalra, 2024; FrameWorks Institute, 2020), FrameWorks Institute (2024) used the Black Lives Matters Movement (BLM) as a case study to demonstrate how narratives, mindsets, and systems are interconnected and can be shaped for social change. The BLM was catalyzed by the increasing number of vigilante and police shootings of unarmed Black boys and men beginning in 2012 with the murder of Trayvon Martin and situated in the long-standing history of state-sanctioned violence toward Black lives. Activists Patrisse Cullors, Alicia Garza, and Opal Tometi, BLM leaders, connected their messages with moral clarity, critique of systems, and community-centered storytelling. Using vehicles such as social media campaigns, documentaries, and philanthropic partnerships, they amplified their core message in both public and political spheres. Between 1995 and 2015, shifts in the public’s thinking about the role of race and racism in the criminal justice system were evidenced by a 30-point increase in Americans who believed the criminal justice system is “biased against Blacks” (pp. 20–22).

As demonstrated by the BLM, narrative change work has the capability of catalyzing change at various levels: personal, collective, and systems/policy action and change. Yet, given this work is a long game strategy that unfolds over years and decades, it can often be difficult to identify and measure clear outcomes as narrative change is “not an end in itself, but a means to an end” (Han & Kalra, 2024, p. 61). In their 2024 report, “Guiding narrative change: Considerations for the philanthropic field,” FrameWorks Institute offered a tool that identified the following four stages with corresponding metrics of progression for assessing narrative change (p. 31):

1. Emergent/Narrative Product: Narrative is expressed in an observable medium. Ways to assess: Narrative outputs, form, and fidelity
2. Developing/Narrative Presence: Narrative is widely available, regularly accessed, and emotionally resonant. Ways to assess: Narrative reach, targets, and reception.
3. Advanced/Narrative Power: Narrative forces a change in decision-making and/or material reality. Ways to assess: Narrative salience, spread, and impact.
4. Achieved/Narrative Dominance: Narrative becomes the dominant narrative, achieving goals for changing mindsets and systems. Ways to assess: Narrative dominance and impacts.

However, FrameWorks Institute (2024) cautions that these metrics of narrative change efforts cannot be fully realized in the absence of narrative instincts (e.g., acknowledgement and articulation of the need for narrative change) and a narrative infrastructure (e.g., a system of resources for effective narrative change) including networks that have been built and maintained. In their landmark Community Violence Action Plan (2024), a network of over 130 leading organizations and individuals in the community violence intervention (CVI) movement recommended narrative change as a critical domain of action toward sustaining and scaling the field of CVI, an evidence-based approach with particular success in reducing violence in Black and Brown communities. There has been increased recent philanthropic investment in narrative change work and narrative infrastructure generation (e.g., Robert Wood Johnson Foundation/ Forward Promise, 2019; Bill & Melinda Gates Foundation, 2019). Most of the existing knowledge about narrative change from conceptualization and process, to why it is effective and how to measure success has largely been led by activists, policy advocates, and philanthropic leaders in partnership with communities to enact social change. Our community-engaged visual storytelling and narrative change campaign is similarly shaped by a multidisciplinary team, philanthropic narrative investments, and shared aspirations to shift narratives and prevent youth violence.

## The Current Project: *In All Ways Human*

Grounded in the *Cycle of Dehumanization* framework, guided by FrameWorks Institute’s (2024) assessing narrative change tool, and building on a decade of community-engaged research on youth violence in Baltimore, the aim of the current project is to disrupt the dehumanizing narrative of Black male criminality through visual storytelling and narrative change. Funded by a 2020 Gates Grand Challenge Award (1 of 28 winners) and a Robert Wood Johnson Foundation production fund, and after securing IRB approval, our multidisciplinary team set out to accomplish this by conducting a photovoice project that centered young Black male survivors of homicide from our previous research (Smith, 2015; Smith & Patton, 2016; Smith Lee & Robinson, 2019; Smith Lee et al., 2020) via a “participatory visual qualitative research method (research that involves direct collaboration with those affected by an issue being studied for the purpose of action or change) that combines photography and narrative storytelling to capture individuals' lived experiences” (Johns Hopkins Center for Health Equity, n.d.). Our hope was to expose root causes of the health inequity of homicide for young Black men in Baltimore to shift the conversation about youth violence toward structural drivers versus individual behaviors.

However, the intersection of the evolving COVID-19 pandemic and expressed participant fears of racialized surveillance and punishment for taking photographs in the community in the wake of George Floyd’s murder during our pilot required us to pivot. In consultation with our community partners and participant-advisors, we pivoted to a community-engaged portrait project that aligned perfectly with our original goals and our envisioned strategy. The result was *In All Ways Human*, a visual storytelling and narrative change campaign on a mission to resist the dominant negative narrative of Black Male Criminality by replacing it with a counter-narrative that proclaims: Black boys, men, and families are always and in all ways human and deserving of dignity, safety, care, and opportunity. We are introducing a counter-narrative with the power to transform the ways in which Black males are seen, see one another, and see themselves. As such, our approach is multilevel and multidimensional, with the potential to disrupt the racial dehumanization that reinforces racism at the structural level. We see this work as fundamental to a long-game strategy of youth violence prevention necessary to shift the *peculiar indifference* about youth violence as it impacts Black communities, catalyze generative investments in youth violence prevention, and facilitate healing mindsets and reactions for Black youth and communities. Photography and storytelling are essential tools that shape cultural narratives systems of knowledge (Han & Kalra, 2024). Guided by best practices in narrative change (FrameWorks, 2024), we leverage a multimodal communications strategy (visual, text, media) to shift cultural mindsets using portraits and stories of Black boys and men in Baltimore, the origin city of the nation’s first ever racial zoning ordinance (Boger, 2009; Power, 1983).

## Method

### Project Team

Our multidisciplinary team is composed of community-engaged and qualitative researchers and trainees in Human Development and Family Studies at UNC Greensboro led by a clinically trained (Marriage and Family Therapist) Principal Investigator (Centering Black Voices Lab Director, Dr. Jocelyn Smith Lee “Dr. J”), a community partner of over 10 years (Historic East Baltimore Community Action Coalition—HEBCAC—Youth Opportunity (YO!) Program; 20-year GED instructor and photographer, Zizwe Allette “Mr. Z”), survivors of youth violence (three past research participants now project participant-advisors: Eric McKnight, Sr., Leon Fountain, III, and Kevin Harvey, Jr.), and an award-winning independent artist and educator (Canadian photographer and trained physician, Zun Lee). We are all homicide survivors and have suffered the loss of loved ones because of youth violence and bring that lived experience to this work. Our team is comprised of both cisgender men and women situated across socioeconomic backgrounds, and all members identify as Black, with one team member (Zun Lee) embodying African American and Korean ancestry.

The P.I., community partner, and participant-advisors share a decade of relational capital and mutual trust that remains foundational to this endeavor. Our aligned values of justice and equity, mutual commitments to improving the health, well-being, and lives of Black boys, men, and families, shared priorities of addressing root causes and catalyzing systems-level change; and, our individual and collective capital are assets that uniquely positioned us to undertake in this narrative change effort. Along with fellow Gates Grand Challenge grantees, we participated in an 18-month Voices for Economic Opportunity Incubator, which equipped us with community and capacity to increase our knowledge about narrative change, identify audience and storytellers, refine and test our ideas, produce narrative outputs, and examine outcomes (FrameWorks Institute, 2024); Han & Kalra, 2024). Our team leveraged our collective strengths in photography, qualitative inquiry, visual artistry, and storytelling toward the generation of narrative change activities and assets.

### Participants

Mindful that the dehumanization and criminalization of Black males begins in childhood and extends posthumously (Lenoard, 2017; Smiley & Fakunle, 2016), we recruited 50 Black boys, teens, and men (ages 2 to 77) to engage with our project. Participation was first extended to current members and alumni of the YO! Center and their children. Snowball recruitment extended participation to community elders and residents of East Baltimore where the HEBCAC YO! Center is situated. Our first portrait sessions began in the Fall of 2021 and they concluded in May of 2022. In addition to a copy of their professional photographs, participants were given a $25 gift card to thank them for their time and offset any costs of participation.

### Procedure

We transformed a communal space of the HEBCAC YO! Center into a pop-up photo and video recording studio, and hosted community portrait days and drop-in sessions when the center reopened its doors, allowing us to photograph and interview in a controlled manner that promoted health and safety as the pandemic evolved. After completing informed consent and signing a project photo release (parents of the three minor participants provided consent and signed photo releases for their children, with children assenting to be photographed), participants completed a portrait session at the HEBCAC YO! Center with Mr. Z and a corresponding brief (30–60 min) virtual qualitative interview with Dr. J. Parents of minors photographed completed interviews about their participating child. No minors were interviewed for this project.

In consultation with our team’s independent artist and educator, Zun Lee, Mr. Z took the portraits. His familiarity and existing relationships with many of the project participants created a sense of connectivity and trust that put participants at ease in front of the camera. Likewise, for the P.I., relational capital from her research addressing the marginalized trauma and grief of young Black men in Baltimore combined with clinical training and expertise in qualitative methods facilitated meaningful semi-structured interviews conducted virtually over Zoom at the time of portrait sessions with participants (and caregivers of participating minors). Interviews centered around two central questions: (1) What is something that is true about you that no one would know simply from looking at you? (2) What do you want the world to know about who you are as a Black boy/man? These organizing questions created space for participants to share their stories and communicate what *they* want the world to know about who they are as human beings. They also facilitated access to our awareness of the dehumanizing stereotypes that participants have encountered across their lives (or fear their sons will encounter for parents of participants). With consultative support from the communication strategists available to us via the Gates Grand Challenge Incubator narrative infrastructure, we developed participant interviews into stories with the form “when people look at me, they assume…when in reality, I..” that captured their answers to interview questions and accompany their portraits in curated physical and digital exhibits. The result is an innovative and viable community engaged intervention strategy centering narrative change in youth violence prevention by disrupting the dehumanizing narrative of Black male criminality.

## Results

**Fig. 1:**
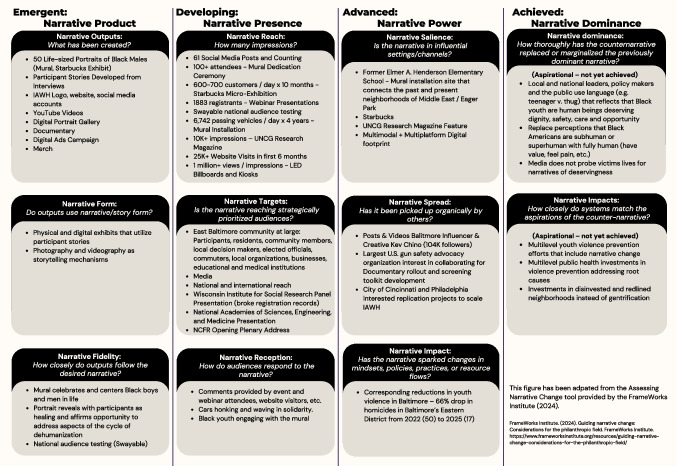
In all ways human (IAWH) assessing narrative change tool

As a direct counterpoint to the mugshot images of Black males that the media widely circulates in print and the daily news circuit, we strategically used photography to capture black and white portraits of 50 intergenerational Black boys and men in Baltimore to visually disrupt the negative narrative of Black male criminality and disseminate our *In All Ways Human* counter-narrative. In the following sections we apply FrameWorks Institute’s (2024) Assessing Narrative Change tool (see Fig. 1 – Smith Lee, 2025) to describe: (1) the progression of our narrative change effort (narrative stages); (2) the outputs we created to express our *In All Ways Human* narrative in observable mediums (Narrative Product); (3) evidence of project reach, audience engagement and narrative reception (Narrative Presence); and, (4) indicators of narrative salience, spread, and impact (Narrative Power). Links are provided for visual engagement with this intervention.

### Emergent Stage: Narrative Product

According to FrameWorks Institute (2024), this stage involves creating narrative outputs, shaping narrative form (e.g., stories), and striving for narrative fidelity across outputs. While the most compelling outputs of our narrative change effort are our photography and story outputs, the expression of our narrative in an observable medium also required more taken for granted processes including identifying “a memorable and recognizable title for the counter-narrative” (FrameWorks Institute, 2024, p. 21), the creation of a project logo, and project branding to help us maintain narrative fidelity across our outputs. Our counter-narrative name, *In All Ways Human*, emerged from a consultative braintrust of the Gates Grand Challenge Incubator in which the P.I. communicated the heart of our teams’ collective impact across community engaged research, health, art, and education as a shared mission to affirm and galvanize systems around the truth that “Black boys, men, and families are always and in all ways human.” This articulation anchored our counter-narrative and focused our outputs as they concertedly work to transform the ways in which Black males are seen, see one another, and see themselves.

### Portraits and Stories

Our fundamental project outputs are the 50 portraits of project participants and their stories that we strategically disseminated in multimodal counter-narrative formats. We began by disseminating these images to the audience most connected and impacted by our work—the participating Black boys and men and their families. We printed each portrait as a life-sized poster (36 × 53) and hosted reveal sessions for each available participant. We also gave smaller prints to participants to keep and share with family. With the support of the Gates Grand Challenge Incubator, individual portraits and stories were juxtaposed side-by-side into postcard-like images consistent with our project branding. Although each participant provided informed consent and a photo release, as our narrative product emerged, we reached out to each participant to get express permission for their photo and story to be shared on our public-facing channels (website/social media accounts). Our digital galleries reflect participant agency.

### Visual Storytelling: Digital Gallery, Physical Exhibits, and Ad Fleets

#### Digital Gallery

The *In All Ways Huma*n digital gallery (https://inallwayshuman.com/in-all-ways-human-always-gallery/) is doing the work of disrupting dehumanization one portrait, one story, at a time. Collectively, these assets are shifting perspectives. The form of our assets (https://www.instagram.com/p/CecwZ0CuVUt/) was decided based on national audience testing available to us through the Gates Grand Challenge Incubator in partnership with data scientists leading the impact testing technology, Swayable. These preliminary findings indicated that portraits that were coupled with participant stories describing a stereotype their life story disrupts were the most effective in shifting negative beliefs about Black boys and men among a nationally representative sample. Swayable testing also helped us confirm that our portraits and stories were effective at increasing the belief that Black males are family-oriented, community-oriented, hardworking, and face more judgment. Evidence of this shift was present across gender, age, income, education level, and political leaning. This early testing helped our team design the online gallery and social media assets, choose images for our Starbucks installation, and strategize our pilot digital ads campaign.

#### Physical Exhibits

Two standing physical exhibits, our *In All Ways Human* Portrait Mural and our Starbucks Micro Exhibition expressed our counter-narrative in public spaces at strategic sites in East Baltimore and with resonance nationally. 

##### Portrait Mural

Using the art and social messaging technique of wheat paste, we installed (https://inallwayshuman.com/baltimore-exhibits/#east-baltimore-portrait-mural) our 50 life-sized posters side-by-side to form a ribbon of intergenerational portraits of Black boys and men spanning the length of a city block on the side of the vacant Elmer A. Henderson Elementary school, half a block away from the HEBCAC YO! Center and two blocks from the medical campus of Johns Hopkins University–a stretch of block situated in a neighborhood revitalization zone where Hopkins employees typically park and walk to work and with high car traffic. This target installation site offered: (1) Proximity to our community partner; (2) Access to critical actors of influence at Johns Hopkins who impact the lives of violence impacted Black male youth (e.g., medical providers and researchers); (3) Visibility to residents of the revitalized community; (4) Physical connectivity between the historic and newly established East Baltimore neighborhoods; and (5) The opportunity to reclaim this site of strategic residential displacement and affirm that economically disadvantaged Black boys, men, and families as *also* deserving of safety, economic mobility, and the full promise of the East Baltimore Development Initiative. Pictured among the participants is an early graduate of that closed school (age 76) and more recent (age 22) student of the school when it was closed in 2007 and designated to become residential units as part of the urban revitalization initiative; one that has simultaneously relocated many economically disadvantaged Black residents.

Our portrait mural sits under 8 giant (200 × 84) vinyl adhesive panels that proclaim: In All Ways Human. Always. The mural stares at a park that is part of a redeveloped parcel of land rebranded Eager Park, where a historically Black East Baltimore neighborhood named Middle East formerly existed. This outdoor exhibit, intentionally designed to promote safe engagement amidst the ongoing pandemic, visually represents the inclusion of boys and men at this site of displacement, inviting residents and powerful local decision makers to embrace Black males as people to be invested in, not social problems to be pushed out, policed, or punished. We designed the *In All Ways Human* mural to be a physical and ideological bridge between these worlds with the potential to transform relationships and outcomes at the intersections of racial justice, gun violence, and economic mobility—which are all physically and visibly situated at this site.

##### Starbuck Micro Exhibition

Our team forged a collaboration with the operator of the Starbucks located two blocks (N. Wolfe St and Ashland Ave.) from our *In All Ways Human* portrait mural who was inspired by our project concept. We installed a micro exhibition at his Starbucks store that is on the first floor of a Science Park/biotech hub in the Eager Park revitalization area and directly across from Johns Hopkins medical campus—two blocks from the HEBCAC YO! Center. This Starbucks exhibit (https://inallwayshuman.com/baltimore-exhibits/#east-baltimore-starbucks-exhibit) featured 10 of our 50 life-sized prints displayed aerially for 10 months in every other pane of glass comprising the store’s edifice.

#### Ad Fleets

Our team launched a local digital ads campaign to disseminate our counter-narrative on jumbo LED Billboards and digital kiosks in downtown Baltimore and the Inner Harbor during Baltimore Orioles homestands. We intentionally placed our portraits in locations where they would be visible to an intersectional (race/class) audience, especially those whose daily lives might not otherwise intersect with Black boys and men. We ran ads on the LED Big Billboard on North Charles Street and on 8 kiosks across the Inner Harbor, Downtown, Camden Yards, Mount Vernon, and Bromo Arts District (https://inallwayshuman.com/baltimore-exhibits/#baltimore-ad-campaign). 

### Website, Social Media, and Merch

To create a central destination where audiences can engage with our counter-narrative and to advance the availability of this narrative change campaign locally, nationally, and globally, we created the *In All Ways Human* website (https://www.inallwayshuman.com) and created social media accounts on X and Instagram (@InAllWaysHuman). These platforms also provided a mechanism for archiving portraits and assessing audience reach. A supplemental production fund award from the Robert Wood Johnson Foundation also enabled us to leverage videography as a narrative output. A clip of our future *In All Ways Human* short documentary can be viewed at https://inallwayshuman.wpengine.com/baltimore-exhibits/#short-documentary.

Responsive to participant-advisor requests and to advance our strategic communications strategy, we produced *In All Ways Human* merch. These outputs include clothing (T-shirts, hoodies, hats), totes, wristbands, buttons, stickers, and water bottles displaying our project name, mission, website, socials, and a QR code for quick engagement with participant portraits and stories, are available during community events and following presentations to help increase the reach, spread, and salience of our narrative change efforts.

### Developing Stage: Narrative Presence

FrameWorks Institute (2024) states this stage involves assessing how many impressions narrative outputs are generating, audience reach, and narrative reception and resonance. In this section, we summarize our insights across outputs (portraits and stories; digital gallery, physical exhibitions, and ad fleets; website, social media, and merch). The response of those centered in this work—Black boys, men, and their families who are most vulnerable to youth violence and racial dehumanization—was evident to us at our portrait reveal sessions. These events created a space for transformative reflection where participants could visualize their own resilience in the face of structural barriers, violence, and unequal burdens of loss, and began to reimagine aloud what is possible for their futures. We observed these moments as healing and an important opportunity to begin addressing the internalized racism and harmful internal feelings described in the C*ycle of Dehumanization*. The impact of these moments was palpable, and snippets can be experienced by visiting https://inallwayshuman.com/our-impact/.

The prospective narrative reception of our counter-narrative became apparent to our team during a test run of the portrait mural when we temporarily affixed sample images to the wall of the vacant school. Cars drove by honking and waving in solidarity. However, it was a small group of tween and teen Black boys who left their play at a nearby park to get a closer look that affirmed the importance of our concept and work. One boy, unaware that his father was part of the project, gazed in awe of his father’s life-sized portrait and began to stand proudly beside it, visually dispelling the dehumanizing myths surrounding Black fathers; delighted to see his dad celebrated in this way (https://www.instagram.com/p/CfIWjzhLyNf/?img_index=1). In a society that predominantly centers Black males in tragedy or violent death, our mural celebrates and centers Black boys and men in life. This is a *mural of the living*. This installation was formally dedicated on June 5, 2022, during Wear Orange Weekend (National Gun Violence Awareness) to strategically connect the issues of racial dehumanization and youth violence for our audiences. The ceremony attracted over 100 family members, friends, and residents in celebration of the humanity of Black boys and men, broadly, and those celebrated in the mural, specifically. Merch was provided to all in attendance, helping us spread our message beyond our project core.

For 10 months (June 2022–April 2023) our micro exhibition in Starbucks accomplished our goal of reaching healthcare professionals, medical trainees, scientists, staff, and patients of the Johns Hopkins medical campus with our *In All Ways Human* counter-narrative. Each day, this Starbucks location services an average of 600–700 patrons. We aimed to daily foreground the humanity of Black boys and men with these critical actors, whose daily interactions with Black males may predominantly be through the delivery of emergency or ambulatory care, especially care resulting from violent injury. One of our participants, an alum of the HEBCAC YO! Baltimore center works in this Science Park as a staff member for a program working to connect Black and Brown youth to data science. To hear him share about the impact of seeing his portrait on display for the first time visit https://www.youtube.com/watch?v=g7ncvK7bHlA. To read his story, visit https://www.instagram.com/p/Ceh2P07OTIi/.

Our website attracted over 25,000 visits to the site in its first 6 months, alone. Data suggests that site visitors come repeatedly, hinting at sustained engagement particularly with our online portrait gallery that showcases participant portraits and stories. These visitors are largely U.S. based; however, they also evidence international reach, with site visitors from the United Kingdom, Canada, Mexico, Brazil, China, India, Russia, Denmark, Ireland, and other European nations. A sample comment left by a site visitor stated: “Awesome! Thank you for showing the humanity of Black men! Racism will cease to exist when everyone sees our humanity.” Our billboard and digital kiosk ad fleets strategically played around Baltimore city generated over 1 million views/impressions over the span of just two weekends. The P.I. has also presented this work on research panels examining resilience among trauma-exposed youth (National Academies of Sciences, Engineering, and Medicine; Wisconsin Institute for Research on Poverty (IRP)—broke IRP webinar registration records signaling high interest), and as the 2025 National Council on Family Relations invited Opening Plenary speaker in Baltimore.

### Advanced Stage: Narrative Power

This narrative stage involves assessing narrative spread, salience, and impact as measured by changes in mindsets, decision-making, and/or material reality (FrameWorks, 2024). It also documents elicited resistance. Indicators of our Narrative Power include the placement of our narrative in influential physical and digital settings/channels including Starbucks and YouTube. Nationally, Starbucks has been a site of criminalization for Black men (The Guardian, 2018). Centering this visual counter-narrative in Starbucks is an intentional narrative change action that reclaims humanity, restores dignity, and reimagines opportunity for Black boys and men.

The UNC Greensboro research magazine featured our project as its cover story in print magazines mailed to over 10 k constituents. Its companion web story was the second most read article of the year. Our labor of love installing the portrait mural was organically captured and posted on social media and YouTube (https://www.youtube.com/watch?v=uuJk5eo8Rt4) by Baltimore Influencer and Creative, Kev Chino (over 104 k followers on Instagram; 15.4 k YouTube subscribers) an indicator of narrative salience and spread. It was also powerfully captured and narrated by a passerby and posted to YouTube (https://www.youtube.com/watch?v=ztyBxVITGZU) where it has gained over 1200 views. In this video posted in September of 2023, the resistance to our narrative was also spontaneously captured. Months following our portrait mural installation, a dehumanizing cycle of heinous vandalism began. Portraits were sliced. Eyes were gouged out from participant portraits, including from the toddler and young children represented in the mural. Words such as domesticated and indoctrinated were sprayed in all caps over the head of portraits. We restored the mural as an act of collective healing on Wear Orange Weekend 2023, again renewing our commitment to advancing the counter-narrative of *In All Ways Human* in the space of violence prevention. Not long after, the mural was vandalized.

In 2024, we changed our installation approach and shifted from the medium of wheat paste to vinyl portraits to increase the durability of the mural and make it harder to slice and remove portraits. It worked. Yet, the resistance grew. In late summer of 2025, red spray paint was used to strike out the eyes of all 50 portraits. The eyes of the youngest child were sprayed out with dots including a singular dot in the center of the forehead mimicking a bullet hole. Following the vandalism, executives overseeing the revitalization zone where the mural is displayed are communicating pressures for its permanent removal. Per FrameWorks (2024), these are all indices of our advancing Narrative Power. Our team is actively mobilizing to fund a sustainable solution to publicly display this powerful portrait mural. Removal of the mural subsequently punishes the pain of our project participants, mirroring the systemic response to youth violence that we are working to disrupt.

Finally, we have received interest from other cities in replicating our project as part of their youth violence prevention efforts. As we strategize to scale and progress to the Achieved stage, our future documentary will yield a narrative output that can be readily implemented nationally and catalyze collaborations for local implementation. We have engaged in preliminary conversations with the largest gun safety advocacy organization in the U.S. about partnering for a national documentary roll out and creating a screening toolkit to engage audience members in reflection about racial dehumanization, youth firearm violence prevention, and calls to action.

## Discussion

We began this paper imploring prevention scientists to critically examine why the nation has failed to systematically respond to the unequal burdens of trauma, injury, and grief resulting from youth violence in the lives of Black Americans (Richardson et al., 2016; Sharpe, et al., 2024; Smith & Patton, 2016; Smith, 2015). We asserted this patterned response reflects historical, contemporary, and fundamental failings to see, care about, protect, and invest in Black people as human beings (not property). Theoretically situated in the *Cycle of Dehumanization* framework (Forward Promise, 2019), we argued that the dominant negative narrative of Black male criminality competes for the will of policy makers and the public to see the pain this health inequity produces in the lives of Black boys, men, and families, activating criminogenic versus public health responses (Leonard, 2017; Sered, 2015). Howard Stevenson (2016) cautions, “by not confronting this historic endorsement of expendable Black humanity, any writing about “novel” approaches for Black male improvement seems futile” (p.57). To this end, we argued that if prevention science aims to address youth violence prevention by addressing structural racism, it must include narrative change interventions as part of its multilevel youth violence prevention efforts, and introduced our visual storytelling and narrative change project, *In All Ways Human*, as a viable solution.

### Strengths

FrameWorks Institute (2024) makes clear that achieving narrative change takes decades. Although just four years in, our *In All Ways Human* project has established a strong foundation of progress: creating our Narrative Product, developing Narrative Presence, and advancing Narrative Power. Our multimodal approach reflects best practice in narrative change (FrameWorks, 2024) and spreads our counter-narrative across diverse outlets aiming to match the widespread approach used to promulgate the narrative of Black male criminality (Fredrickson, 1971). Preliminary evidence indicates our counter-narrative can shift mindsets about the ways Black boys and men are seen (Swayable testing), disrupting negative stereotypes anchored in dominant negative narratives, and centering humanity. While not yet at the Achieved stage, as we strive for Narrative Dominance, our aspiration is that leaders, policy makers and the public, locally and nationally, would respond to the health inequity of youth violence with language (e.g., teenagers vs. thugs) that reflects a baseline perception that Black youth are human beings worthy of dignity, safety, care, and opportunity. We also aspire to system alignment with our counter-narrative, with public and political will favoring multilevel public health investments in violence prevention addressing root causes over criminal justice approaches. The landmark 2025 CVI report named narrative change as a critical dimension of realizing this shift, and our *In All Ways Human* project offers a timely, innovative, and viable strategy to help realize this goal.

Preliminary evidence also indicates that our counter-narrative can impact the ways Black boys and men see one another and see themselves. Affirming the humanity of Black males across the life course (Smith Lee, 2016) and foregrounding their resilience (Walker et al., 2024) are critical healing actions for Black males impacted by youth violence that can further disrupt the harmful internal feelings and external reactions named in the Cycle of Dehumanization. In particular, photography and qualitative interviewing are valuable methodological tools in this work. Researchers, clinicians, and youth programmers can all critically consider how to leverage these strategies in their efforts to prevent youth violence, and we aspire to produce a toolkit that can provide technical assistance for adoption and implementation of *In All Ways Human*.

### Limitations and Future Directions

Despite its contributions, our narrative change effort is not without limitations. Our project builds on a decade of community engaged research with young Black male homicide survivors; however, we know dehumanizing racial narratives impact Black Americans of multiple and varied intersecting identities that increase their vulnerability to the health inequities of violence. As we aspire to narrative dominance, we envision a future in this work where our *In All Ways Human* counter-narrative expands and is adopted and adapted to promote health and well-being for more Black Americans.

We also see promising opportunities to scale this healing work in other cities (Mehranbod et al., 2022) where the relationship between firearm violence and structural racism has been empirically identified, with the cities of Cincinnati and Philadelphia expressing early interest in replication. Beginning this work in Baltimore is significant because of the historic promulgation of negative racial narratives city leaders leveraged to invent the first racial zoning ordinances in this nation (Boger, 2009; Power, 1983). Given Baltimore’s focus in the literature on structural racism (redlining) and firearm violence (Uzzi et al., 2023), we envision future collaborative research that tests and evaluates *In All Ways Human* as a structural approach to firearm violence intervention. Likewise, we are eager to empirically examine the mechanisms by which our intervention may enhance the existing CVI ecosystem in Baltimore or directly contribute to youth violence prevention in the neighborhoods in which it is embedded. For example, while Baltimore data show a 66 percent drop in homicides in Baltimore’s Eastern district where our portrait mural is situated from 2022 (year of *In All Ways Human* launched) to the present (The Baltimore Sun, 2025), a reduction that mirrors the city’s overall historic drop in homicides, we do not currently have the capacity to test this relationship. We are committed to understanding how our work may advance the ongoing CVI efforts in the city to reduce and sustain reductions in youth homicide. Baltimore has made significant and successful public health investments in CVI and violence prevention that “match the aspirations of [our *In All Ways Human*] counter-narrative” (FrameWorks Institute, 2024, p. 31). Therefore, *In All Ways Human* is well-positioned to work creatively alongside existing CVI interventions, while doing the critical work of disrupting the dehumanizing narratives that too often reinforce the health inequities of youth and firearm violence.

## Conclusion

Preventing youth firearm violence through structural and community-engaged approaches that address racism also requires addressing the peculiar indifference (Du Bois,^1899^; Loren Dreir, 2021) of the nation to the epidemic of gun violence that has unequally buffeted Black youth and families (Smith, 2015). *In All Ways Human* is one example of a viable, theoretically driven, multi-level solution that aims to address racial dehumanization as a fundamental root cause that both creates contexts of vulnerability to youth violence and maintains the inequity Black youth face in experiencing it. As we witness contemporary racial dehumanization across American cities, the work of narrative change in violence prevention is both timely and critical.

## Data Availability

The data that support the findings of this study are not publicly available due to privacy or ethical restrictions.
